# Clinical characteristics of impulse control and related disorders in Chinese Parkinson’s disease patients

**DOI:** 10.1186/s12883-017-0874-6

**Published:** 2017-05-18

**Authors:** Yu Zhang, An qi He, Lin Li, Wei Chen, Zhen guo Liu

**Affiliations:** Department of Neurology, Xinhua Hospital Affiliated to Shanghai Jiao Tong University School of Medicine, 1665 Kong jiang Road, Shanghai, 200092 People’s Republic of China

**Keywords:** Impulse control and related disorders, Parkinson’s disease, Chinese

## Abstract

**Background:**

Impulse control and related disorders (ICRDs) are clinically complications in Parkinson’s disease (PD). However, the clinical characteristics of ICRDs in Chinese PD patients were rarely reported. We aimed to explore the prevalence and the clinical profile of ICRDs in Chinese patients with PD.

**Methods:**

142 Chinese PD patients were consecutively enrolled. The symptoms of ICRDs were assessed with the Questionnaire for Impulsive-Compulsive Disorders. The clinical characteristics of patients with ICRDs and without ICRDs were compared.

**Results:**

ICRDs were present in 31% of our patients. The most common ICRDs were compulsive medication use (11.3%) and punding (9.2%); the least frequent were walkabout (1.4%). Variables independently associated with ICRDs were earlier onset of the disease (≤55 years), severe cognitive impairment (MMSE 10–20), the dose of dopamine agonist (>1 mg/d) and dyskinesia.

**Conclusions:**

ICRDs was commonly found in Chinese PD patients. Earlier onset of the disease, the dose of dopamine agonist, severe cognitive impairment and dyskinesia are independent factors associated with ICRDs. Our results will be benefit for clinicians to assess the risk of developing ICRDs before delivering dopaminergic medication.

## Background

Impulse control and related disorders (ICRDs) were defined as a number of behaviors involving repetitive, excessive and compulsive activities that interferes in major areas of life functioning [1]. Pathological gambling(PG), hypersexuality(HS), compulsive buying(CB) and binge eating(BE) were major symptoms of ICRDs. Other ICRDs are composed of punding, hobbyism, walkabout and compulsive medication use [1]. As a relatively recent addition to the behavioral spectrum of Parkinson’s disease (PD) related non-motor symptoms, ICRDs are becoming increasingly concerned.

The prevalence of ICRDs in PD was reported by different studies in different population, ranging from3.5% to 42.8% [2, 3]. These studies showed many factors may play a role in the risk of ICRDs in PD patients, including the use of dopamine agonists (DA), male gender, young patient, depression, smoking, drug abuse, genetic factors, family history of ICRDs and so on [4–6]. Especially, dopamine agonists were thought to be the major predictors for ICRDs [6]. Recently, Questionnaire for Impulsive-Compulsive Disorders in Parkinson’s disease (QUIP) was developed and widely used to screen ICRDs in PD patients [7]. It is well known that, Chinese PD patients are typically treated with lower dosages of medications and present cultural differences in perceptions of disease [8]. This raises the question that whether the prevalence of ICRDs is lower in Chinese PD patients and which factors affect the risk of ICRDs in Chinese PD patients. Therefore, our study aims to study the prevalence and the possible impact factors of ICRDs in Chinese PD patients.

## Methods

### Participants

142 subjects were enrolled at the department of neurology, Xin Hua Hospital affiliated to Shanghai Jiaotong University School of Medicine from December 2014 to October 2016. Patients were diagnosed as PD according to the UK Brain Bank criteria [9]. The records of PD medication and dosage were taken at the time of assessment. Patient with secondary Parkinsonism, deep brain stimulation, stroke, brain tumor and treated with levodopa infusion were excluded. Once those patients included in the study, a structured interview for clinical and demographic variables was performed by a neurologist. All participants had written informed consent. This study was performed with the approval of the Ethics Committee of Xin Hua Hospital affiliated to Shanghai Jiaotong University School of Medicine.

### Clinical assessment

The following demographic and clinical features data were retrieved from subjects: gender, age, education,alcoholism and smoking status, age at PD onset, duration of disease, predominant symptoms at PD onset, the history of dopamine replacement therapy and related complications (Total levodopa equivalent daily dose, LEDD, was calculated according to previously suggested conversion formulae) [10], Hoehn-Yahr stage (H&Y), Unified Parkinson’s disease Rating Scale (UPDRS), the scale for freezing of gait, Minimum Mental State Examination (MMSE), non-motor symptom (NMS), REM Sleep Behavior Disorder Questionnaire Hong Kong (RBDQ-HK), Hamilton Anxiety Scale (HAMA), Hamilton Depression Scale (HAMD), and Parkinson’s Disease Questionaire-39 (PDQ-39). The symptoms of ICRDs were assessed with QUIP.

### Statistical analysis

Statistical analysis was performed using SPSS 20.0; Categorical variables were compared using the Pearson’s Chi-Square test or the Mann-Whitney test or Student’s t-test; Binary logistic regression model was performed to evaluate factors that could independently associate with ICRDs. Level of significance was set at *p* < 0.05. Odds ratio (OR) are presented with their 95% confidence intervals (95% CI).

## Results

### Subject characteristics

142 Chinese PD patients participated in our study. Demographic and clinical information of our patients are listed in Table 1. Of them, 73 (51.4%) were male and 69 (48.6%) were female. Their mean age at study was 68.39 ± 8.14 years, mean age at onset of PD motor symptoms was 62.06 ± 9.27 years and mean disease duration was 6.00 ± 5.56 years. The mean MMSE score was 26.18 ± 4.07 and the mean Hoehn-Yahr stage was 2.25 ± 0.84 (stage 1 and 1.5, *n* = 41; stage 2 and 2.5, *n* = 69; stage 3, *n* = 21, and stage 4 and 5, *n* = 11). The mean UPDRS motor score for the entire study group was 19.32 ± 12.42.The mean total UPDRS score for the entire group was 33.23 ± 19.06, the mean UPDRS IV(motor complication) score was 0.99 ± 2.27,and the mean UPDRS 32 + 33(dyskinesia)score was 0.29 ± 1.00. Antiparkinsonian drugs with DA was reported in 49.3% (DA-LEDD 41.90 ± 52.13) and L-dopa in 68.3% (LEDD 314.09 ± 320.63) of patients. The total LEDD was 389.40 ± 373.72. The most common medication was a combination of levodopa and DA, which was used by 63 (44.4%) of the patients, with 10 of them using additionally a MAO-B inhibitor. The combination of DA and MAO-B inhibitor was used by 10 (7%) patients. Monotherapy was used by 25 (17.6%) of the patients (19 (13.4%) levodopa, 6 (4.2%) DA). None of our patients was reported to have family history of impulse control disorders.

**Table 1:** Characteristics of Chinese PD patients with and without ICRDs

		Mean ± SD		
Characteristic	All(*n* = 142)	Without ICRDs(*n* = 98)	ICRDs Positive(*n* = 44)	*P* value
Male,%	51.4	55.1	43.2	0.208
Age at study,y	68.39 ± 8.14	69.67 ± 8.16	65.55 ± 7.43	0.005
Age of PD onset,%				<0.001
≤55y	24.6	15.3	45.5	
56-60y	16.2	16.3	15.9	
>60y	59.2	68.4	38.6	
PD duration,y	6.00 ± 5.56	5.22 ± 5.23	7.76 ± 5.90	0.010
Current Smoking,%	21.1	16.3	31.8	0.037
Current Alcoholism,%	16.2	18.4	11.4	0.336
L-dopa medication,%	68.3	64.3	77.3	0.172
DA medication,%	49.3	40.8	68.2	0.003
Total LEDD,mg	389.40 ± 373.72	329.82 ± 340.65	522.06 ± 412.46	0.006
L-dopa LEDD,%				0.061
≤250 mg	43.7	48.0	34.1	
250-500 mg	32.4	33.7	29.5	
>500 mg	23.9	18.4	36.4	
DA LEDD,%				<0.001
≤50 mg	63.4	71.4	45.5	
51-100 mg	25.4	24.5	27.3	
>100 mg	11.3	4.1	27.3	
L-dopa treatment duration,y	3.89 ± 5.25	3.42 ± 5.21	4.98 ± 5.23	0.042
DA treatment duration,y	1.93 ± 3.00	1.44 ± 2.32	3.03 ± 3.93	0.020
H&Y stage	2.25 ± 0.84	2.21 ± 0.77	2.32 ± 0.99	0.727
UPDRS Total	33.23 ± 19.06	32.17 ± 19.32	35.59 ± 18.46	0.208
UPDRS I	3.17 ± 1.77	3.13 ± 1.79	3.25 ± 1.77	0.710
UPDRS II	9.61 ± 6.22	9.34 ± 5.97	10.23 ± 6.79	0.666
UPDRS III	19.32 ± 12.42	18.93 ± 12.82	20.18 ± 11.56	0.390
UPDRS IV	0.99 ± 2.27	0.57 ± 1.39	1.93 ± 3.36	0.020
UPDRS 32 + 33	0.29 ± 1.00	0.11 ± 0.66	0.68 ± 1.46	0.001
Freezing of gait	4.10 ± 7.41	2.96 ± 6.40	6.64 ± 8.85	0.010
MMSE score, %				0.049
10–20	12.0	8.2	20.5	
21–26	27.5	25.5	31.8	
≥27	60.6	66.3	47.7	
NMS score	27.51 ± 22.07	27.29 ± 23.67	28.02 ± 18.24	0.318
HAMA score	6.49 ± 5.30	5.83 ± 5.15	7.95 ± 5.40	0.020
HAMD score	7.63 ± 7.50	7.53 ± 7.58	7.84 ± 7.39	0.657
RBDSQ-HK score	18.15 ± 15.78	17.36 ± 16.20	19.93 ± 14.83	0.192
PDQ-39 score	20.28 ± 18.28	19.95 ± 18.19	21.02 ± 18.66	0.752

*Abbreviations*: *LEDD* levodopa equivalent daily dose, *UPDRS* Unified Parkinson’s disease Rating Scale, *MMSE* Minimum Mental State Examination, *NMS* non-motor symptom, *HAMA* Hamilton Anxiety Scale, *HAMD* Hamilton Depression Scale, *RBDSQ-HK* REM Sleep Behavior Disorder Questionnaire Hong Kong, *PDQ-39* Parkinson’s Disease Questionaire-39

3.2. Frequency of ICRDs.

**Fig. 1:**
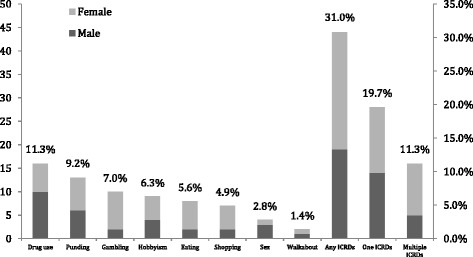
Prevalence of ICRDs clinical subtypes and gender distribution

The frequencies and clinical features of ICRDs screening with QUIP are illustrated in Fig. 1.We identified 44(31.0%) patients with ICRDs (28 presented 1 ICRDs, and 16 presented multiple ICRDs). compulsive medication use (11.3%) was the most frequent, followed by punding (9.2%), pathological gambling (7.0%), hobbyism (6.3%), binge eating (5.6%), compulsive buying (4.9%), hypersexuality (2.8%) and walkabout (1.4%). Male patients are prone to report compulsive medication use and hypersexuality.

### 3.3. Association between ICRDs and clinical variables

Compared with PD patients without any ICRDs, ICRDs positive patients were younger, earlier onset of PD, and had a longer disease course. More smokers and lower MMSE score were found in patients with any ICRDs. Then, ICRDs patients were more likely to have higher scores in UPDRS IV, UPDRS 32 + 33(dyskinesia), HAMA and the scale for freezing of gait. In ICRDs group, the DA-LED was 61.61 ± 61.37 mg/d while the DA-LED in non-ICRDs group was much lower (30.36 ± 42.97 mg/d). The doses of levodopa did not show any difference between two groups. However, there was no statistically significant difference in gender, marriage status, education background, the PD phenotype at PD onset, depression, quality of life, RBD, non-motor symptoms, UPDRS or H&Y stage between two groups.

Variables entered into the multiple logistic regression analysis were: age at PD onset, DA LEDD, UPDRS 32 + 33 score and L-dopa treatment duration, after adjusting for all the covariates that were associated with ICRDs (sex, age, age of onset, smoking, TLEDD,DA-LEDD, levodopa dosage and the MMSE, HAMA, UPDRS 32 + 33, freezing of gait) in multivariable analysis. In the analysis, earlier onset of the disease (*p* = 0.001,OR 5.99,95%CI 2.10–17.09), the dose of oral DA LEDD (p<0.001, OR 15.94,95%CI 3.66–69.45), lower MMSE score(p=0.002, OR 6.71,95%CI 2.03–22.17)and dyskinesia (UPDRS32 + 33 score)(*p* = 0.04,OR 4.94,95%CI 1.07–19.99) were significantly increased the odds of ICRDs (Table 2).

**Table 2:** Multivariate binary logistic regression analyses of the factors associate with ICRDs

Variables	OR (95% CI)	*P* value
Age of PD onset≤55y	5.99(2.10–17.09)	0.001
Age of PD onset 56-60y	2.02(0.58–7.01)	0.266
DA LEDD 51-100 mg	1.50(0.53–4.21)	0.442
DA LEDD >100 mg	15.94(3.66–69.45)	<0.001
Dyskinesia	4.63(1.07–19.99)	0.040
MMSE 10–20	6.71(2.03–22.17)	0.002
MMSE 21–26	1.34(0.48–3.77)	0.576

## Discussion

Identification of ICRDs in PD patients will be benefit for clinicians ﻿to making﻿ therapeutic decisions and avoiding important social, economic and legal problems for patients. In our study, nearly one third of our subjects present at least one kind of ICRDs (31%), which is similar in some population, such as Malaya (35%) [11], India (31.6%) [3], Finland (34.8%) [5] and Denmark (35.9%) [12]. However, the incidence of phenotypes of ICRDs was diverse in different population. In our patients, the most common ICRDs were compulsive medication use (11.3%). On the contrary, the most frequent ICRDs was PG in North America (DOMINION study) [6], punding in India [3], hypersexuality in Finland [5], hobbyism and punding in Denmark [11]. The cause of these differences may be that the questionnaire used for screening ICRDs in those studies were different with ours. On the other hand, cultural, social and economic factors might also play a role on the phenotype of ICRDs in Chinese PD patients (e.g., casino is illegal and patients were ashamed to report sexual behavior to doctor in China). The overall prevalence of ICRDs was similar among male and female in this study, however, there are some slightly differences exist in the incidence of phenotypes of ICRDs, HS was more frequency in male, but BE, PG and CB was more frequency in female. Our results indicated QUIP has high sensitivity, and patients need to be evaluate the risk of developing ICRDs when exposure to dopaminergic medication. Deep brain stimulation is rarely implemented in Chinese PD patient. Furthermore, the mechanisms of deep brain stimulation inducing impulsive controlling disorders might be different with drug induced impulse control disorders. Therefore, we exclude those patients treated with deep brain stimulation and will investigate the role of deep brain stimulation on ICRDs in future.

Consistent with previously studies [6], usage of DA is correlates with ICRDs among Chinese patients with PD. We did not carry on the sub-analysis of dopamine subtype,especially of D3 receptor stimulus agonist, since only pramipexole and piribedil are available in China. In addition, only 6 of our patients took piribedil, sub-analysis was not suitable for our study. Furthermore, the regression analyses suggest that the dosages of DA (>1 mg/d) was the strongest association factor for ICRDs, so we should be more careful in the course of increasing the dose of DA.

In addition, our study indicated that dyskinesia was independently associated with ICRDs. There might be many reasons for this result. Firstly, from common risk factor perspective, both ICRDs and dyskinesia might be affected by excessive dopaminergic drug stimulation, earlier onset of the disease and severe cognitive impairment [13, 14]. Secondly, from pathophysiology perspective, both ICRDs and dyskinesia might be induced by excessive stimulation in the motor or limbic territories of the striatum. But the detail mechanism of biochemical, molecular and functional changs need further investigation. These findings remind clinicians that youth patients with PD who intake high dose of dopamine agonist and have poor cognitive function should be prevent from both ICRDs and dyskinesia.

The relatively serious cognitive impairment appeared in PD patients with ICRDs (measured by MMSE) in our study. It may be caused by impaired top-down inhibitory control from prefrontal cortical area. However, the association between cognition and ICRDs in PD subjects is still debatable according to previous studies [12, 15, 16]. Moreover, freezing of gait was also related to ICRDs, even though it did not enter the regression model. It consist with PD patients have gait disturbance were more prone to developed ICRDs which have been reported in other studies [17]. The limitations of our investigation may be from the recruitment of subjects from a single movement disorder center, the lack of an age matched control group, cross-sectional evaluation at a single time point, the small sample size and the questionnaire we used.

## Conclusions

ICRDs are common in Chinese PD patients. QUIP was a useful screening instrument for ICRDs in PD. Early onset of PD, dyskinesia and dopamine replacement therapies were significantly associated with ICRDs. Clinicians need to routine elevate the risk of developing ICRDs before delivering dopaminergic medication, especially those with certain clinical and demographic characters, such as earlier onset of the disease, cognitive impairment, higher dosage of DA and dyskinesia.

## Abbreviations

ICRDs: Impulse control and related disorders
PD: Parkinson’s disease
PG: Pathological gambling
HS: Hypersexuality
CB: Compulsive buying
BE: Binge eating
DA: Dopamine agonists
QUIP: Questionnaire for Impulsive-Compulsive Disorders in Parkinson’s disease
H&Y: Hoehn-Yahr stage
UPDRS: Unified Parkinson’s disease Rating Scale
MMSE: Minimum Mental State Examination
NMS: Non-motor symptom
RBDQ-HK: REM Sleep Behavior Disorder Questionnaire Hong Kong
HAMA: Hamilton Anxiety Scale
HAMD: Hamilton Depression Scale
PDQ-39: Parkinson’s Disease Questionaire-39
OR: Odds ratio
LEDD: Levodopa equivalent daily dose
